# Association of maternal serum lipids at late gestation with the risk of neonatal macrosomia in women without diabetes mellitus

**DOI:** 10.1186/s12944-018-0707-7

**Published:** 2018-04-11

**Authors:** Xiangxiang Wang, Qingbo Guan, Jiajun Zhao, Feifei Yang, Zhongshang Yuan, Yongchao Yin, Rui Fang, Lingwei Liu, Changting Zuo, Ling Gao

**Affiliations:** 1 Department of Endocrinology, Shandong Provincial Hospital affiliated to Shandong University, Jinan, Shandong 250021 China; 2 Shandong Clinical Medical Center of Endocrinology and Metabolism, Jinan, Shandong 250021 China; 3 Institute of Endocrinology and Metabolism, Shandong Academy of Clinical Medicine, 544, Jing 4 Rd, Jinan, Shandong 250021 China; 4 Department of Biostatistics, School of Public Health, Shandong University, Jinan, Shandong 250012 China; 5 Department of Scientific Research, Shandong Provincial Hospital affiliated to Shandong University, Jinan, Shandong 250021 China; 6 Department of Obstetrics and Gynecology, Shandong Provincial Hospital affiliated to Shandong University, Jinan, Shandong 250021 China; 7 Scientific Center, Shandong Provincial Hospital affiliated to Shandong University, 544, Jing 4 Rd, Jinan, Shandong 250021 China

**Keywords:** Lipids, Triglyceride, Macrosomia, Birth weight, Pregnancy

## Abstract

**Background:**

Macrosomia is a serious public health problem worldwide due to its increasing prevalence and adverse influences on maternal and neonatal outcomes. Maternal dyslipidemia exerts potential and adverse impacts on pregnant women and newborns. However, the association between maternal serum lipids and the risk of macrosomia has not yet been clearly elucidated. We explored the association between the maternal lipids profile at late gestation and the risk of having macrosomia among women without diabetes mellitus (DM).

**Methods:**

The medical records of 5407 pregnant women giving birth to single live babies at term were retrospectively analyzed. Subjects with DM, hypertension, thyroid disorders and fetal malformation were excluded. Maternal fasting serum lipids were measured during late pregnancy. Logistic regression analysis was used to analyze the variables associated with the risk of macrosomia.

**Results:**

Maternal serum triglyceride (TG) and high-density lipoprotein cholesterol (HDL-C) levels were related to macrosomia; each 1 mmol/L increase in TG resulted in a 27% increase in macrosomia risk, while each 1 mmol/L increase in HDL-C level resulted in a 37% decrease in macrosomia risk, even after adjusting for potential confounders. Notably, the risk of macrosomia increased progressively with increased maternal serum TG levels and decreased HDL-C levels. Compared with women with serum TG levels < 2.5 mmol/L, women with TG levels greater than 3.92 mmol/L had an approximately 2.8-fold increased risk of macrosomia. Compared with women with serum HDL-C levels above 2.23 mmol/L, women with HDL-C levels of less than 1.62 mmol/L had a 1.9-fold increased risk of giving birth to an infan with macrosomia. In addition, a higher risk of macrosomia was observed in women with simultaneous hypertriglyceridemia and low serum HDL-C levels (odds ratio [OR] 2.400, 95% confidence interval [CI]: 1.760–3.274) compared to those with hypertriglyceridemia or low serum HDL-C alone (OR 2.074, 95% CI: 1.609–2.673 and OR 1.363, 95% CI: 1.028–1.809, respectively).

**Conclusions:**

Maternal serum TG levels and HDL-C levels at late gestation are independent predictors of macrosomia in women without DM.

**Electronic supplementary material:**

The online version of this article (10.1186/s12944-018-0707-7) contains supplementary material, which is available to authorized users.

## Background

Macrosomia, defined as a fetal birth weight equal to or greater than 4000 g, irrespective of gestational age, has a serious impact on maternal and fetal perinatal outcomes [1, 2]. Mothers delivering infants with macrosomia are at a higher risk of prolonged labor, caesarean section, postpartum haemorrhage, and perineal trauma. For infants, macrosomia is associated with an elevated risk of shoulder dystocia, clavicular fracture, perinatal asphyxia, brachial plexus injury, and perinatal mortality [3]. Furthermore, children born with macrosomia are more likely to suffer obesity, diabetes mellitus (DM), early cardiovascular disease, and certain cancers later in life [4–7]. In recent decades, an increasing prevalence of fetal macrosomia has been reported in both developed countries and developing countries [8, 9]. Therefore, it is of great importance to prevent adverse pregnancy outcomes by exploring the risk factors for macrosomia.

Until now, a variety of factors, such as high maternal age, height, pre-gestational body mass index (BMI), gravidity, excessive gestational weight gain, post-term pregnancy and male fetal sex, have been associated to increased risks of macrosomia [1, 3, 8, 10, 11]. Above all, maternal metabolism affects the development and growth of the fetus. Hyperglycemia is a well-established metabolic factor that may result in an adverse intrauterine environment and ultimately lead to fetal overgrowth [12–14]. Maternal lipid profile can also induce excessive fetal growth [15, 16]. The changes in lipid metabolism during pregnancy are characterized by fat accumulation in maternal depots during early pregnancy and development of hyperlipidemia later [17]. Recently, some maternal lipid parameters have been identified as independent risk determinants of fetal overgrowth, especially in pregnancies complicated by GDM or with DM [15, 18]. Several studies have demonstrated that in non-diabetic pregnancies, birth weight and the risk of macrosomia or large for gestational age (LGA) were positively associated with maternal serum triglyceride (TG) levels [16, 19, 20], and negatively associated with high-density lipoprotein cholesterol (HDL-C) levels [21], While other studies failed to find any association [22, 23]. Studies have shown that patterns of maternal dyslipidemia and the prevalence of macrosomia vary across populations and ethnic groups [23, 24]. While a few studies have explored the relationship between maternal serum lipids and neonatal birth weight in Chinese population, their sample sizes were relatively small [21, 25].

In the present study, we sought to evaluate the association between maternal serum lipid levels at late gestation and the risk of macrosomia among a large group of Chinese women without DM. Moreover, we assessed the combined impact of maternal serum TG and HDL-C on macrosomia risk.

## Methods

### Study design and groups

Pregnant women who were admitted to Shandong Provincial Hospital affiliated to Shandong University and delivered between January 2013 and September 2015 were recruited for the present study. We established the study cohort based on inclusion and exclusion criteria. The inclusion criteria were as follows: 1) live-born singleton pregnancy; 2) delivery at 37–42 gestational weeks; 3) naturally conceived; and 4) had integrated medical records and a clear gestational age. We excluded women with pre-gestational DM or gestational diabetes mellitus (GDM), hypertensive disorder, and thyroid disorder to eliminate the influences of these diseases on lipid metabolism and infant birth weight. Subjects who delivered before 37 weeks of gestation as well as cases of fetal congenital malformation or multifetal gestation were also excluded. In addition, we excluded those who had delivered neonates with a birth weight of less than 2500 g since they are prone to unfavorable perinatal outcomes.

Finally, 5407 pregnant women from 18 to 48 years of age at 37 to 42 weeks of gestational age were selected and enrolled in the final analysis. This study was approved by the Ethics Committee of Shandong Provincial Hospital, and written informed consent was obtained from every participant before enrollment.

### Data collection

Data were extracted from medical records, including maternal age, height, weight before delivery, blood pressure, family history of DM among first-degree relatives, gestational age at delivery, gravidity, mode of delivery, metabolic parameters, neonatal birth weight, birth length, and neonatal sex. Weight and height were measured in kilograms and centimeters, respectively, and BMI was calculated by dividing the weight in kilograms by the square of the height in meters. Gestational age was calculated based on the last menstrual period, as confirmed by ultrasonography. Maternal metabolic parameters included fasting plasma glucose (FPG), serum TG, total cholesterol (TC), HDL-C, low-density lipoprotein cholesterol (LDL-C), and serum uric acid (UA). Blood samples were drawn between 37 and 42 weeks of gestation to measure metabolic parameters after overnight fasting. All biomedical analyses were performed using an automatic biochemical analyzer (Olympus AU5400, Tokyo, Japan) at Shandong Provincial Hospital.

### Statistical analysis

All statistical analyses were conducted using SPSS statistical software (version 23.0 for Windows; SPSS Inc., Chicago, IL, USA). Normally and non-normally distributed continuous variables are presented as mean ± standard deviation and median (interquartile range), respectively, and categorical variables are presented as number (percentage). Differences between groups were tested using the independent two-sample t test, the Mann-Whitney test, and the Chi-squared test. Logistic regression analysis was performed to identify risk factors that were significantly correlated with macrosomia. Maternal systolic blood pressure (SBP), BMI before delivery, FPG, gestational age, and infant sex, which were statistically significant according to univariate logistic regression, were regarded as confounding variables in the multivariable logistic regression model. Serum lipids were entered into model 1 individually and into model 2 together. A family history of DM was further adjusted in model 3. All statistical tests were two-tailed, and *p* values < 0.05 were considered significant.

## Results

### Characteristics of the study population

Maternal and neonatal characteristics are displayed by birth weight in Table 1. Of the 5407 women, 541 delivered macrosomia newborns and 4866 delivered normal birth weight newborns. The latter were regarded as controls. The overall prevalence of macrosomia was 10.0%**.** The median of serum TG levels in the macrosomia group was much higher than that in the control group (3.52 mmol/L vs. 3.09 mmol/L, *p* < 0.001), while the serum HDL-C and LDL-C levels were lower in the macrosomia group (1.85 ± 0.45 vs.1.96 ± 0.48, p < 0.001; 3.18 ± 0.96 vs. 3.36 ± 1.01, p < 0.001, respectively). There was no significant difference in TC levels between the two groups. In addition, women who delivered an infant with macrosomia were taller, heavier, had higher BMI, higher SBP, higher FPG levels, and longer gestational age at delivery than the controls. The occurrence of macrosomia was markedly higher in boys than in girls. Moreover, caesarean delivery was more frequent in women delivering an infant with macrosomia than in women giving birth to a baby of normal birth weight (56.3% vs. 45.2%, *p* < 0.001). Maternal age, diastolic blood pressure (DBP), UA, a family history of DM, and gravidity of the neonates in the two groups were similar (Table 1).

**Table 1:** Maternal and neonatal characteristics

	Control	Macrosomia	*P* value
Number	4866	541	
Maternal characteristics			
Age (years)	29.91 ± 3.89	29.98 ± 3.70	0.679
Height (cm)	162.69 ± 4.59	164.56 ± 4.63	0.000
Weight (kg)	73.32 ± 9.42	79.99 ± 9.95	0.000
BMI (kg/m2)	25.92 ± 7.47	28.01 ± 7.29	0.000
SBP (mmHg)	120.07 ± 12.25	121.30 ± 11.67	0.028
DBP (mmHg)	76.63 ± 9.33	77.44 ± 8.95	0.054
TC (mmol/L)	6.71 ± 1.31	6.64 ± 1.29	0.222
HDL-C (mmol/L)	1.96 ± 0.48	1.85 ± 0.45	0.000
LDL-C (mmol/L)	3.36 ± 1.01	3.18 ± 0.96	0.000
TG (mmol/L)	3.09 (1.39)	3.52 (1.72)	0.000
FPG (mmol/L)	4.35 ± 0.44	4.44 ± 0.46	0.000
UA (mmol/L)	278.92 ± 68.73	277.20 ± 65.15	0.579
Family history of DM (number, %)	135 (2.8%)	17 (3.1%)	0.624
Neonatal characteristics			
Male sex (number, %)	2453 (50.4%)	329 (60.8%)	0.000
Birth weight (g)	3381.02 ± 327.55	4219.98 ± 230.55	0.000
Birth length(cm)	49.39 ± 1.63	51.61 ± 1.45	0.000
Gravidity			0.460
1	2411 (49.5%)	259 (47.9%)	
> 1	2455 (50.5%)	282 (52.1%)	
Delivery mode			0.000
Vaginal delivery	2665 (54.8%)	237 (43.7%)	
Caesarean section	2201 (45.2%)	304 (56.3%)	

All data are expressed as mean ± standard deviation, median (interquartile range) or number (percentage). Abbreviations: BMI, body mass index; SBP, systolic blood pressure; DBP, diastolic blood pressure; TC, total cholesterol; HDL-C, high-density lipoprotein cholesterol; LDL-C, low-density lipoprotein cholesterol; TG, triglyceride; FPG, fasting plasma glucose; UA, uric acid; DM, diabetes mellitus

### Maternal serum TG and HDL-C levels are associated with macrosomia risk

We first investigated whether there was an association between maternal serum lipids and the risk of having an infant with macrosomia. Using univariate logistic regression analysis, we found that the risk of macrosomia increased approximately 1.245-fold with each 1 mmol/L increase in TG, while the risk decreased by 41% or 17% with each 1 mmol/L increase in HDL-C or LDL-C, respectively (Table 2). Meanwhile, we identified other confounders that were correlated with the presence of macrosomia in addition to serum lipids and found that maternal SBP, BMI before delivery, FPG, gestational age, and infant sex were statistically significant (Table 2). Therefore, these variables were regarded as confounders in the multivariable logistic model. When maternal serum lipids were entered individually into model 1, TG and HDL-C were still greatly associated with macrosomia risk after adjusting for confounding factors; each 1 mmol/L increase in TG led to a 27% increase in macrosomia risk, and each 1 mmol/L increase in HDL-C level led to a 37% decrease in macrosomia risk. When maternal serum TG, HDL-C, and LDL-C were entered together into model 2, we also found that maternal TG and HDL-C levels were related to an increased or decreased risk of macrosomia (odds ratio [OR] 1.249, 95% confidence interval [CI]: 1.176–1.326, per 1 mmol/L increase and OR 0.770, 95% CI: 0.612–0.970, per 1 mmol/L increase, respectively) (Table 3). However, no association was observed between TC or LDL-C and macrosomia risk in the multivariate model. Maternal BMI before delivery, FPG, gestational age, and infant sex were also statistically significant in the multivariable logistic model (see Additional file 1: Table S1). Further adjustment for family history of DM had little effect on the models (see Additional file 1: Table S2). These findings show that serum TG levels and HDL-C levels are independent predictive risk factors of macrosomia in non-diabetic women.

**Table 2:** Univariate binary logistic regression of risk factors for macrosomia

Variables	B	SE	OR	95%CI for OR	*P* value
TG	0.219	0.026	1.245	1.183–1.311	0.000
TC	−0.043	0.035	0.958	0.894–1.026	0.223
HDL-C	−0.519	0.101	0.595	0.488–0.726	0.000
LDL-C	−0.185	0.047	0.831	0.757–0.912	0.000
Age	0.004	0.012	1.004	0.981–1.027	0.742
SBP	0.008	0.004	1.008	1.001–1.015	0.029
DBP	0.009	0.005	1.009	1.000–1.019	0.060
BMI	0.148	0.014	1.160	1.130–1.191	0.000
FPG	0.411	0.102	1.508	1.236–1.840	0.000
Family history of DM	0.128	0.261	1.137	0.681–1.898	0.624
Gestational age	0.432	0.044	1.541	1.414–1.680	0.000
Male sex	0.423	0.093	1.527	1.273–1.830	0.000
Gravidity	0.012	0.056	1.012	0.907–1.131	0.827

Data are coefficient (B), standard error (SE.), odds ratio (OR), 95% confidence interval (CI) and significance (P value). Abbreviations: TG, triglyceride; TC, total cholesterol; HDL-C, high-density lipoprotein cholesterol; LDL-C, low-density lipoprotein cholesterol; SBP, systolic blood pressure; DBP, diastolic blood pressure; BMI, body mass index; FPG, fasting plasma glucose; DM, diabetes mellitus

**Table 3:** Multivariate logistic regression analyses of maternal lipids and macrosomia risk

	B	SE	OR	95% CI for OR	*P* value
Multivariate Model* (model 1)					
TG	0.241	0.030	1.273	1.200–1.350	0.000
TC	0.035	0.040	1.036	0.957–1.121	0.381
HDL-C	−0.464	0.113	0.628	0.504–0.784	0.000
LDL-C	−0.097	0.053	0.907	0.818–1.007	0.067
Multivariate Model* (model 2)					
TG	0.222	0.031	1.249	1.176–1.326	0.000
HDL-C	−0.261	0.118	0.770	0.612–0.970	0.026
LDL-C	−0.038	0.053	0.962	0.868–1.068	0.470

Data are coefficient (B), standard error (SE), odds ratio (OR), 95% confidence interval (CI) and significance (P value).* The multivariate model was adjusted for maternal systolic blood pressure, body mass index, fasting blood glucose, gestational age at delivery, and fetal sex. Model 1: maternal lipid levels were entered into the modelindividually; model 2: maternal TG, HDL-C, and LDL-C levels were entered into the model together. Abbreviations: TG, triglyceride; TC, total cholesterol; HDL-C, high-density lipoprotein cholesterol; LDL-C, low-density lipoprotein cholesterol

### Macrosomia risk increases progressively with higher serum TG levels and lower serum HDL-C levels

**Fig. 1:**
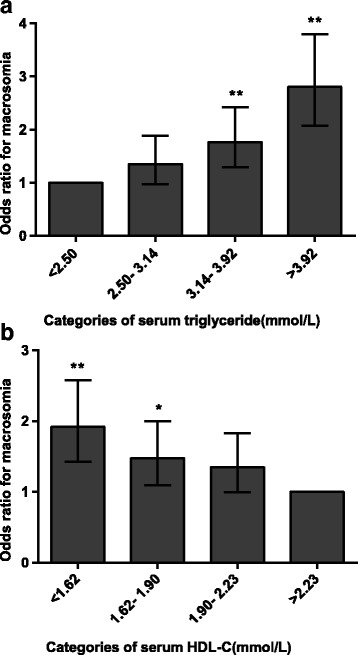
Correlation of serum triglyceride levels (**a**) and HDL-C levels (**b**) with macrosomia risk. The data are expressed as the odds ratio (OR) and 95% confidence interval (CI). *p < 0.05, ** *p* < 0.01, vs. the group with serum triglyceride levels less than 0.97 mmol/L or vs. the group with serum HDL-C levels greater than 2.23 mmol/L. The error bars represent the 95% CI. Abbreviations: HDL-C, high-density lipoprotein cholesterol

To determine the presence of macrosomia across the spectrum of serum TG levels or serum HDL-C levels, we stratified TG and HDL-C into four groups (quartiles) for the entire study population. With elevated serum TG or decreased HDL-C levels, the risks macrosomia gradually increased after adjustment for potential confounding factors. Compared with the group with serum TG levels of less than 2.5 mmol/L, the risk of macrosomia was markedly increased (approximately 2.8-fold) when TG levels were greater than 3.92 mmol/L (Fig. 1a). Similarly, women in the lowest quartile of HDL-C (< 1.62 mmol/L) had a 91.9% increased risk of delivering an infant with macrosomia compared with those in the highest quartile of HDL-C (≥2.23 mmol/L) (Fig. 1b). These results demonstrate that serum TG levels and HDL-C levels are related to macrosomia risk in a dose-dependent manner.

### Maternal hypertriglyceridemia increases the risk of macrosomia particularly in the presence of low serum HDL-C levels

**Fig. 2:**
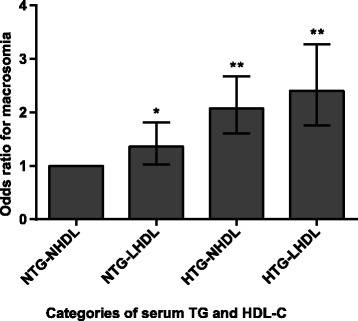
Correlation of hypertriglyceridemia and low HDL-C levels with macrosomia risk. The NTG-NHDL group (normal TG combined with normal HDL-C) was used as the reference group. The data are expressed as the odds ratio (OR) and 95% confidence interval (CI). *p < 0.05, ** p < 0.01 vs. the NTG-NHDL group. Abbreviations: NTG: normal triglyceride; HTG: high triglyceride; NHDL: normal high-density lipoprotein cholesterol; LHDL: low high-density lipoprotein cholesterol

Maternal hypertriglyceridemia was defined as levels higher than the 75th percentile (> 3.92 mmol/L) of all subjects. Low serum HDL-C level was defined as levels lower than the 25th percentile (< 1.62 mmol/L) of all subjects. We identified the individual and combined contributions of hypertriglyceridemia and low serum HDL-C to the risk of macrosomia. We divided the study objects into four subgroups according to TG and HDL-C levels as follows: 1) a NTG-NHDL group with normal serum TG and normal HDL-C levels; 2) a NTG-LHDL group with normal TG levels and low HDL-C levels; 3) a HTG-NHDL group with hypertriglyceridemia and normal HDL-C levels; and 4) a HTG-LHDL group with hypertriglyceridemia and low HDL-C levels. The NTG-NHDL group was regarded as the control. We analyzed the differences in macrosomia risk among the four groups. As presented in Fig. 2, macrosomia risk was positively correlated with the presence of hypertriglyceridemia and low serum HDL-C alone (OR 2.074, 95% CI: 1.609–2.673 and OR 1.363, 95% CI: 1.028–1.809, respectively); the predictive effect of hypertriglyceridemia on macrosomia was stronger than that of low serum HDL-C levels. Furthermore, the presence of hypertriglyceridemia and low HDL-C levels together was correlated with the greatest risk of macrosomia (OR 2.400, 95% CI: 1.760–3.274). These results demonstrate that both maternal hypertriglyceridemia and low HDL-C were independently correlated with macrosomia risk. Their combination had a greater predictive value than either alone.

Published studies evaluating the associations between maternal serum lipids at different gestation periods and neonatal birth weight or the risk of macrosomia/LGA (Table 4).

**Table 4:** Published studies evaluating the associations between maternal serum lipids and neonatal birth weight

First author, year	N	Range of GA at sampling (weeks)	State of blood sampling	GDM included	Major findings	Ref
Wang, 2016	5218	4–13	NF	Yes	Low TG level was a protective factor for LGA, while high LDL-C level was a risk factor for macrosomia. After adjusting for confounders, no significant associations were found between lipid levels and macrosomia or LGA.	[40]
Vrijkotte, 2011	4162	13 (12–14)^*^	NF	No	The highest TG level was associated with a higher BWSDS and a higher prevalence of LGA than the middle quintile.	[16]
Vrijkotte, 2012	4008	13 (12–14)^*^	NF	No	Elevated TG levels were associated with an increased risk of LGA.	[29]
Parlakgumus, 2014	433	< 14	F	No	No lipids were correlated with fetal birth weight.	[23]
Clausen, 2005	2050	17–19	NF	Yes	High non-HDL-C and low HDL-C levels were associated with increased an risk of macrosomia.	[30]
Liu, 2016	1546	24–28	F	Yes	Neonatal birth weight was associated with TG levels.	[31]
Sommer, 2015	699	28	F	Yes	HDL-C was a predictor of birth weight.	[32]
Cianni, 2004	83	27 ± 3.7^#^	F	No	TG levels were independently associated with neonatal birth weight.	[19]
Kitajima, 2001	146	24–32	F	No	TG levels were correlated positively with newborn weight at term.	[20]
Mossayebi, 2014	154	25–32	F	No	TG was an independent predictor of birth weight, LGA, and macrosomia.	[34]
Retnakara, 2012	472	30 (28–32)*	F	No	None of the lipid measurements was independently associated with birth weight or the risk of LGA.	[22]
Hou, 2014	2790	28–37	F	No	High maternal TG levels were significantly associated with LGA newborns.	[35]
Ye, 2015	1243	36–41	F	No	HDL-C was independently associated with neonatal size and was an independent predictor for LGA.	[21]
Jin, 2016	934	7–10 21–24 33–37	F	Yes	High TG in late pregnancy was independently associated with increased risks of LGA and macrosomia. Relatively low HDL-C levels during pregnancy were associated with an increased risk of macrosomia.	[25]
Kulkarni, 2013	631	18 ± 2,^#^ 28 ± 2^#^	F + NF	Yes	TG was associated with birth weight at 28 weeks (unadjusted). TC at both 18 and 28 weeks were independently associated withbirth weight.	[12]

*The data are expressed as median (interquartile range); # The data are expressed as mean ± standard deviation. Abbreviations: GA, gestational age; F, fasting; NF, non-fasting; GDM, gestational diabetes mellitus; TG, triglyceride; TC, total cholesterol; HDL-C, high-density lipoprotein cholesterol; LDL-C, low-density lipoprotein cholesterol; BWSDS, birth weight standard deviation score; LGA, large for gestational age; Ref, reference

## Discussion

In our hospital-based cross-sectional study, we noted that maternal serum TG concentrations in late pregnancy were independently and positively associated with the risk of macrosomia in women without DM, whereas there was a negative correlation with HDL-C levels. These associations were dose-dependent. Furthermore, an increased macrosomia risk was correlated with maternal hypertriglyceridemia or low HDL-C levels and a more obvious association was observed in mothers with both hypertriglyceridemia and low HDL-C levels. These findings suggest that maternal serum TG and HDL-C levels in the third trimester may be strong predictors of macrosomia risk.

Recently, risk factors of macrosomia have received considerable attention because of its adverse influences on maternal and neonatal complications. Several risk factors have been described in previous studies, including older maternal age, greater height, higher BMI, excessive gestational weight gain, post-term pregnancy, pre-gestational DM and GDM, male fetal sex, and higher gravidity. In the present study, we found that maternal BMI before delivery, FPG, gestational age at delivery, and infant sex were independently associated with the risk of macrosomia.

Fetal growth is affected by metabolic factors of the mother. Maternal glucose is a well-known risk factor for fetal overgrowth in women with DM [13, 26]. Moreover, the Hyperglycaemia and Adverse Pregnancy Outcomes (HAPO) study showed graded linear increases in LGA across the entire distribution of glucose in women with glucose levels below the diagnostic threshold for DM [14]. Furthermore, an increased incidence of LGA infants and macrosomia was found in women with abnormal screening for GDM but normal glucose tolerance test results [27]. In consistent with these results, we found that maternal FPG at late gestation was an independent risk factor for macrosomia, suggesting that maternal FPG had an important role in fetal overgrowth even in non-diabetic women. A family history of DM is related to an increased risk of GDM or overt DM. Subjects with a family history of DM are prone to insulin resistance [28]. Therefore, we also observed the effect of family history of DM on macrosomia risk, but no significant effect was found. Moreover, further adjustment for a family history of DM had little effect on other potential risk factors in our study.

Maternal lipid metabolism is another vital determinant of fetal development and growth. Whether abnormal maternal lipid metabolism in non-diabetic pregnancies contributes to fetal overgrowth remains controversial at this stage (as shown in Table 4). Maternal lipid profile at early and mid-pregnancy gestation is significantly associated with neonatal birth weight, and with the risk of macrosomia or LGA [16, 29–32]. However, the association seems more complicated for late-stage gestation. Retnakaran et al. reported that none of maternal lipids was independently associated with birth weight or the risk of LGA [22]. Another study found that neonatal birth weight was inversely associated with HDL-C at late gestation only in overweight/obese women [33]. Mossayebi et al. argued that Maternal TG was an independent predictor of neonatal birth weight, LGA and macrosomia in non-diabetic, non-obese pregnant women [34]. Consistent with our results, previous studies conducted in Chinese women showed that high TG and/or low HDL-C levels in late pregnancy were independently associated with increased risks of macrosomia and LGA [21, 25, 35]. In the present study, we further reported that these associations were dose-dependent, i.e. higher maternal TG levels and lower maternal HDL-C levels resulted in a higher prevalence of macrosomia. While Kulkarni et al. have reported a positive correlation between maternal TC levels and neonatal birth weight [12], no significant association was detected between maternal TC or LDL-C levels and birth weight in our study.

To further separately identify the contributions of abnormal maternal serum TG and HDL-C to macrosomia, we observed the effect of hypertriglyceridemia or low HDL-C levels individually on the risk of macrosomia. We found that an increased risk of macrosomia was correlated with the presence of either hypertriglyceridemia or low serum HDL-C levels alone. Furthermore, we expanded insights into the combined predictive value of hypertriglyceridemia and low serum HDL-C levels to macrosomia by using these factors as a joint risk assessment tool and found that using both factors together predicted higher risk than either alone. That is, adding low HDL-C levels to hypertriglyceridemia could clearly improve the ability to predict macrosomia. Thus, the combination of hypertriglyceridemia and low HDL-C in the third trimester was a stronger predictor of macrosomia risk in women without DM.

Although significant associations between maternal TG or HDL-C levels and newborn weight were demonstrated, the mechanisms have not yet been fully elucidated. Maternal serum TG does not directly cross the placenta, but the presence of lipoprotein receptors, various fatty acid-binding proteins, and lipase activity in the placenta enables the release of free fatty acids (FFAs) from TG and their efficient transport to the fetus. It is speculated that the increased hydrolysis of maternal TG by placental lipoprotein lipase to FFAs and the excessive delivery of fatty acids to the fetus may be partly responsible for the increased risk of macrosomia among women with hypertriglyceridemia. Furthermore, Wang et al. suggested that enhanced insulin resistance might link dyslipidemia and fetal overgrowth [36]. HDL-C plays an important role in cholesterol transport and homeostasis, which may have an impact on fetal development. Fetal cholesterol is obtained endogenously by de novo synthesis and exogenously by transfer of maternal cholesterol through the yolk sac and/or placenta. Studies in the Golden Syrian hamster have shown that the transport of maternal cholesterol to the fetus is affected by maternal plasma cholesterol concentrations [37]. In addition, HDL-C might affect fetal metabolism and growth via its effect on the metabolic function of extra-embryonic fetal tissues, as demonstrated by a study that revealed that a difference in maternal HDL-C concentration or composition can affect the size of the fetus and sterol metabolism of the yolk sac and placenta in the mouse [38]. Furthermore, HDL-C has anti-inflammatory, antioxidant, and antithrombotic properties that might influence placental circulation and fetal growth [39]. Despite the possible mechanisms mentioned above, these do not fully explain the associations between TG or HDL-C and macrosomia. Much work is still needed to clearly explain how maternal lipids affect fetal growth.

In our study, we systematically explored the risk of macrosomia across the spectrum of maternal lipid levels. Our large sample size may provide more reliable information and improve the power of this study. In addition, combining maternal serum TG and HDL-C as a joint risk assessment tool to predict macrosomia risk represents a novel perspective of our study.

The retrospective nature is the main limitation of our study. First, selection and information bias cannot be totally excluded. Second, causal relationships cannot be completely established by retrospective studies. Finally, several potential confounding factors, such as maternal gestational weight gain, pre-gestational BMI, and prediabetes, were missing in the present study. Prediabetes, characterized by abnormal glucose metabolism, is associated with dyslipidemia [28]. Maternal prediabetic status may affect the relationship between maternal lipids and fetal growth. So far, few studies have assessed the relationship between maternal serum lipids and neonatal growth independent of prediabetes. Prospective studies are needed to elucidate the effect of prediabetes on fetal growth.

## Conclusion

In conclusion, our results revealed that high maternal serum TG concentrations and low maternal HDL-C levels in late pregnancy are independently associated with an increased risk of macrosomia in women without DM. The combination of hypertriglyceridemia and low HDL-C levels was a stronger predictor of macrosomia than either alone. Our findings highlight the importance of maternal lipid metabolism in fetal overgrowth and may have implications for the etiology and primary prevention of macrosomia. However, further prospective investigations involving larger population and basic research studies are necessary to fully evaluate their clinical value and the mechanisms involved.

## Additional file

Additional file 1: **Table S1.** Multivariate logistic regression analysis of other risk factors for macrosomia. **Table S2.** Multivariate logistic regression analysis after furthur adjusted for family history of DM. (DOCX 20 kb)

## Abbreviations

BMI: Body mass index
CI: Confidence interval
DBP: Diastolic blood pressure
DM: Diabetes mellitus
FFAs: Free fatty acids
FPG: Fasting plasma glucose
GDM: Gestational diabetes mellitus
HDL-C: High-density lipoprotein cholesterol
LDL-C: Low-density lipoprotein cholesterol
LGA: Large for gestational age
OR: Odds ratio
SBP: Systolic blood pressure
TC: Total cholesterol
TG: Triglyceride
UA: Uric acid
