# Supplementary material for: Association of maternal serum lipids at late gestation with the risk of neonatal macrosomia in women without diabetes mellitus
Source: Lipids Health Dis. 2018 Apr 11;17:78. doi: 10.1186/s12944-018-0707-7 (PMC5896067; doi:10.1186/s12944-018-0707-7)
Supplement: Supplementary file 1 — Table S1. Multivariate logistic regression analysis of other risk factors for macrosomia. Table S2. Multivariate logistic regression analysis after furthur adjusted for family history of DM. (DOCX 20 kb) [file 12944_2018_707_MOESM1_ESM.docx]

**Additional file 1:**

**Table S1** Multivariate logistic regression analysis of other risk factors for macrosomia

|  | **B** | **SE** | **OR** | **95% CI for OR** | **P value** |
| --- | --- | --- | --- | --- | --- |
| **Multivariate Model* (model 2)** |  |  |  |  |  |
| SBP | -0.002 | 0.004 | 0.998 | 0.990-1.006 | 0.642 |
| BMI | 0.143 | 0.015 | 1.154 | 1.127-1.189 | 0.000 |
| FPG | 0.341 | 0.115 | 1.406 | 1.122-1.762 | 0.003 |
| Gestational age | 0.487 | 0.049 | 1.628 | 1.478-1.792 | 0.000 |
| Male sex | 0.560 | 0.104 | 1.751 | 1.427-2.148 | 0.000 |

Data are coefficient (B), corresponding standard error,(SE.), odds ratio (OR), 95% confidence interval (CI) and significance (P value).*The multivariate model was adjusted for maternal systolic blood pressure, body mass index, fasting blood glucose, gestational age at delivery and fetal sex. Model 2: maternal triglyceride, high-density lipoprotein cholesterol, low-density lipoprotein cholesterol were entered into the model together. Abbreviations: SBP, systolic blood pressure; BMI, body mass index; FPG, fasting plasma glucose; DM, diabetes mellitus.

**Table S2** Multivariate logistic regression analysis after furthur adjusted for family history of DM

|  | **B** | **SE** | **OR** | **95% CI for OR** | **P value** |
| --- | --- | --- | --- | --- | --- |
| **Multivariate Model* (model 3)** |  |  |  |  |  |
| TG | 0.222 | 0.031 | 1.249 | 1.176-1.327 | 0.000 |
| HDL | -0.260 | 0.118 | 0.771 | 0.612-0.971 | 0.027 |
| LDL | -0.038 | 0.053 | 0.962 | 0.868-1.068 | 0.469 |
| SBP | -0.002 | 0.004 | 0.998 | 0.990-1.006 | 0.645 |
| BMI | 0.143 | 0.015 | 1.154 | 1.121-1.189 | 0.000 |
| FPG | 0.340 | 0.115 | 1.406 | 1.122-1.761 | 0.003 |
| Gestational age | 0.487 | 0.049 | 1.628 | 1.478-1.793 | 0.000 |
| Male sex | 0.560 | 0.104 | 1.750 | 1.426-2.147 | 0.000 |
| Family history of DM | 0.049 | 0.294 | 1.050 | 0.590-1.868 | 0.869 |

Data are coefficient (B), corresponding standard error,(SE.), odds ratio (OR), 95% confidence interval (CI) and significance (P value).*The multivariate model was adjusted for maternal systolic blood pressure, body mass index, fasting blood glucose, gestational age at delivery and fetal sex; model 3: further adjusted for family history of DM. Abbreviations: TG, triglyceride;; HDL-C, high-density lipoprotein cholesterol; LDL-C, low-density lipoprotein cholesterol; SBP, systolic blood pressure; BMI, body mass index; FPG, fasting plasma glucose; DM, diabetes mellitus.
